# GCNCMI: A Graph Convolutional Neural Network Approach for Predicting circRNA-miRNA Interactions

**DOI:** 10.3389/fgene.2022.959701

**Published:** 2022-08-05

**Authors:** Jie He, Pei Xiao, Chunyu Chen, Zeqin Zhu, Jiaxuan Zhang, Lei Deng

**Affiliations:** ^1^ School of Computer Science and Engineering, Central South University, Changsha, China; ^2^ Department of Electrical Engineering, University of California, San Diego, San Diego, CA, United States

**Keywords:** circRNA, miRNA, deep learning, graph convolution neural network, circRNA-miRNA interaction

## Abstract

The interactions between circular RNAs (circRNAs) and microRNAs (miRNAs) have been shown to alter gene expression and regulate genes on diseases. Since traditional experimental methods are time-consuming and labor-intensive, most circRNA-miRNA interactions remain largely unknown. Developing computational approaches to large-scale explore the interactions between circRNAs and miRNAs can help bridge this gap. In this paper, we proposed a graph convolutional neural network-based approach named GCNCMI to predict the potential interactions between circRNAs and miRNAs. GCNCMI first mines the potential interactions of adjacent nodes in the graph convolutional neural network and then recursively propagates interaction information on the graph convolutional layers. Finally, it unites the embedded representations generated by each layer to make the final prediction. In the five-fold cross-validation, GCNCMI achieved the highest AUC of 0.9312 and the highest AUPR of 0.9412. In addition, the case studies of two miRNAs, hsa-miR-622 and hsa-miR-149-5p, showed that our model has a good effect on predicting circRNA-miRNA interactions. The code and data are available at https://github.com/csuhjhjhj/GCNCMI.

## 1 Introduction

Non-coding RNA (ncRNA) refers to various RNA molecules that will not translate into a protein. There has been much agreement through numerous studies that ncRNA has monumental biological functions though it only part a small fraction of the genomes. Since the discovery of RNA and ribosomal RNA in the 1950s, non-coding RNA that plays a biological role has been known for 60 years (Palazzo and Lee, 2015). As well as their roles at the transcriptional and post-transcriptional levels, ncRNA plays a critical role in epigenetic regulation of gene expression. The recent finding suggests that some of these RNAs are also involved in translation and splicing (Steitz and Moore, 2003; Butcher and Brow, 2005; Gesteland et al., 2006).

MicroRNA (miRNA) was discovered in 1993 by the Ambros and Ruvkun groups in *Caenorhabditis elegans* (Lee et al., 1993) and brought a revolution to molecular biology. They are small single-stranded molecules that derive from transcripts’ unique hairpin structures called pre-miRNA. Most miRNAs are transcribed from DNA sequences into primary miRNAs, then processed into precursor miRNAs and become mature miRNAs finally (O’Brien et al., 2018; Liu et al., 2021). Furthermore, miRNAs have been found to regulate gene expression post-transcriptionally by affecting mRNA translation, implying that dysregulation of miRNAs may be associated with various diseases by affecting gene expression (Bartel, 2004). For instance, recent studies showed approximately 50% of annotated human miRNAs are located in cancer-associated regions of the genome called fragile sites. This indicated that miRNA plays a crucial role in cancer progression (Calin et al., 2004).

Circular RNA consists of large non-coding RNAs produced by a non-canonical splicing event called back splicing. They are ubiquitous in species ranging from viruses to mammals during post-transcriptional processes. Viroids are the first circRNA to be discovered, though they are not produced by a back splicing mechanism (Sanger et al., 1976). A few years later, most circRNAs are observed in the cytoplasm and some small fractions in the nucleus. Circular forms of RNAs were observed or synthesized in diverse species such as viruses (Kos et al., 1986), prokaryotes (Ford and Ares, 1994), unicellular eukaryotes (Grabowski et al., 1981), and mammals (Capel et al., 1993). Most circRNA are expressed from known encoding proteins, composed of single or multiple exons. With the progress of high-throughput RNA-sequencing and bioinformatics tools, scientists have found the human transcriptome’s general feature ubiquitous in many other metazoans.

A diverse set of circRNAs have been identified as having functions such as sponges, decoys, or translatable elements that alter gene or protein expression. Biological functions of circRNAs have only been investigated for a small fraction, while most of which are proposed as miRNA sponges (Hansen et al., 2013; Memczak et al., 2013; Deng et al., 2022). Sponging up miRNA and interacting with RNA-binding proteins (RBP), circRNA plays many pathological functions like regulating miRNA activity. He et al. (He et al., 2022) performed circRNA microarray analysis and found its expression profile in diabetes. By acting as microRNA sponges for miR-7 (ciRS-7) and miR-124-3p and miR-338-3p (circHIPK3), ciRS-7 and circHIPK3 promote insulin secretion. circRNAs were identified in cancers, so it also proposed to play a crucial role in the intimation and development of tumors. (Ashwal-Fluss et al., 2014; Dong et al., 2017; Soslau, 2018). Most studies focus on the role of circRNA in tumors. circRNA was described as oncogenes. Diverse cellular functions of circRNA suggest their potential for cancer treatment as biomarkers and therapeutic targets (Chen and Huang, 2018; Li et al., 2019).

The interactions between circRNA and miRNA have been gradually discovered in recent years, and some related databases have been established. The CircR2Cancer database (Lan et al., 2020) contains 1,439 interactions between 1,135 circRNAs and 82 cancers. In addition, the database also includes basic information such as detection methods and expression patterns of circRNAs. However, there are few datasets on direct circRNA-miRNA interactions. Moreover, the known interactions are only a tiny part. Discovering the interactions between circRNAs and miRNAs is beneficial to understanding the interactions between circRNA and miRNA and disease. Using biological experiments to verify the interactions between circRNA-miRNA is time-consuming and labor-intensive. Computational methods can be used to mine the interactions between circRNA-miRNA more effectively. Still, there is little work to predict the circRNA-miRNA interactions.

As far as we know, GCNCMI is the first method to predict the circRNA-miRNA interactions, but other methods in the field of bioinformatics are still worth reference. Many methods based on computational interactions have recently achieved good results in predicting microbe-disease interactions and ncRNA-disease interactions. AE-RF (Deepthi and Jereesh, 2021) build an autoencoder to mine potential interaction features and then train a random forest model to predict circRNA-disease interactions. The DMFMDA (Liu et al., 2020) uses one-hot encoding of diseases and microorganisms to convert a vector representation in a low-dimensional space by embedding the propagation layer. The obtained vector representation is then input into a multi-layer neural network, and the parameters of the neural network are continuously optimized through Bayesian sorting to achieve accurate prediction. Deng et al. (Deng et al., 2020) constructed a meta-pathway-based circRNA-disease feature vector. This vector representation combines multiple similarities such as circRNA similarity, disease similarity, etc. The prediction is finally achieved using a random forest classifier. KATZHMDA (Chen et al., 2017) predicts the interactions between unknown microbes and disease by the Gaussian kernel similarity between known microbes and disease. NTSHMDA (Luo and Long, 2018) constructs a disease-microbe heterogeneity network based on the known similarity between microorganisms and diseases and assigns equal weights to known disease-microbe interactions according to the different contributions of diseases and microorganisms, which is conducive to reducing prediction error. Liu et al. (Dayun et al., 2021) established a multi-component graph attention network, which first passed a decomposer to identify node-level feature vectors, then combined the feature vectors to obtain a unified embedding vector, which was finally input into a fully connected network to predict microorganisms unknown interactions with the disease. SDLDA (Zeng et al., 2020) extract the linear and nonlinear interactions between lncRNA and diseases through singular value decomposition and neural network and finally unites the linear and nonlinear features into a new feature vector, which is input to the fully connected layer to realize prediction.

Although the above methods have achieved good prediction results, there are still some problems that will affect mining efficiency. Some existing association prediction methods rely on known similarities, but it is difficult to construct such similarities with the increasing number of miRNAs and circRNAs. There are far fewer known associations than unknown associations. Therefore, these methods are unsuitable when the circRNA and miRNA data increase. When the scale of data increases, how to mine the higher-order interactions of circRNA-miRNA is an urgent problem to be solved. In this paper, we construct a bipartite graph to describe the interaction information between circRNA and miRNA using known relationship pairs of them. Then we develop a graph convolutional network method to mine the deep semantic information that carries collaborative signal in the bipartite graph. We propagate the information flow recursively over the graph structure and continuously aggregate the interactive information between nodes to refine the embedding of each node. Finally, We concatenate the embeddings generated by each layer to predict the relationship of unknown circRNA-miRNA pairs. Experimental results show that our GCNCMI model outperforms the other six state-of-the-art methods.

## 2 Materials and Methods

### 2.1 Datasets

We built the benchmark dataset from the circBank database (Liu et al., 2019). circBank contains 140,790 circRNAs. Each circRNA collects information such as miRNA binding sites, protein-coding ability, etc. We removed redundant parts of the dataset and extracted 2,115 circRNAs and 821 miRNAs from the circBank database, including 9,589 known circRNA-miRNA interactions. It now can be downloaded on the website http://www.circbank.cn/downloads.html. In addition, we randomly selected 9,589 unlabeled samples from the benchmark dataset. The detailed information can be seen in Table 1.

**TABLE 1:** The number of circRNAs, miRNAs, and circRNA-miRNA interactions included in the dataset.

circRNA	miRNA	interactions	unlabled interactions
2,115	821	9,589	9,589

### 2.2 Problem Description

Our work aims to predict unknown relationships based on known circRNA and miRNA relationships. We use $U=\left\{u_{1},u_{2}\ldots u_{n}\right\}$ and $V=\left\{v_{1},v_{2},\ldots ,v_{m}\right\}$ to respectively represent the collection of n circRNAs and m miRNAs, and use the interaction matrix $R\in R^{n\times m}$ to represent the relationship between them. If the circRNA *u*_*i*_ is related to miRNA *v*_*j*_, then the *R*_*ij*_ = 1, otherwise *R*_*ij*_ = 0. It should be noted that *R*_*ij*_ = 0 can only indicate that the two RNAs have not yet found a relationship, but may actually be related.

### 2.3 Graph Construction

**FIGURE 1:**
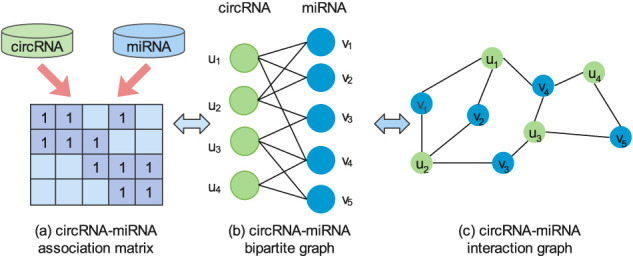
An illustration of the circRNA-miRNA association matrix **(A)**, a bipartite graph **(B)**, and an interaction graph **(C)**.

We use a bipartite graph G(U ∪ V, E) constructed by the interaction matrix $R\in R^{n\times m}$ to show the relationship between circRNAs and miRNAs, where *U*, *V* are the vertex sets denoting the circRNAs and miRNAs, and E is the edge sets constructed from the association matrix $R\in R^{n\times m}$ . This bipartite graph can be expanded into a complex interaction graph as shown in Figure 1. This interaction graph contains the higher-order interaction information of circRNA and miRNA, from which we can mine deep semantic information that carry collaborative signal. For example, the path *u*_1_ − *v*_1_ − *u*_2_ and *u*_1_ − *v*_2_ − *u*_2_ indicate the behavior similarity between *u*_1_ and *u*_2_, as both circRNAs have interacted with *v*_1_ and *v*_2_. Then, the interaction between *u*_2_ and *v*_3_ suggests that *u*_1_ and *v*_3_ are likely to be related.

### 2.4 GCNCMI

**FIGURE 2:**
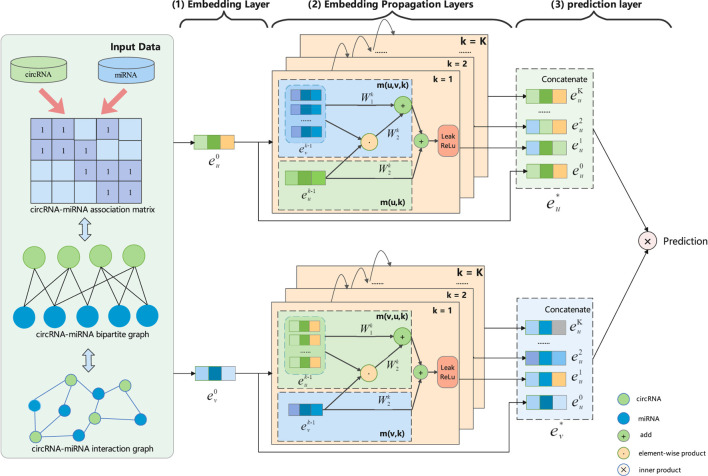
An illustration of GCNCMI model architecture (the arrowed lines present the flow of information). Using GCNCMI to predict the relationship between circRNA u (green) and miRNA v (blue) mainly includes three steps: (1) In the embedding layer, we use input data to initialize circRNA embedding $\left(e_{u}^{0}\right)$ and miRNA embedding $\left(e_{v}^{0}\right)$ ; (2) In embedding propagation layers, the embeddings are continuously refined by recursively aggregating higher-order interaction information; (3) In the prediction layer, we concatenate the embeddings from different propagation layers and make the final prediction.

To capture the deep interaction information embedded in the interaction graph, we model the high-order interaction information of circRNA-miRNA in the embedding function. We propagate the information flow recursively over the graph structure and continuously aggregate the information of neighboring nodes to refine the embedding representation of the nodes (Hamilton et al., 2017; Xu et al., 2018; Wang et al., 2019). The architecture of our proposed GCNCMI model is shown in Figure 2. There are three parts to the framework: 1) An embedding layer that offers initialized circRNA embeddings and miRNA embeddings from the input data; 2) multiple embedding propagation layers that refine the embeddings by aggregating higher-order interaction information; 3) the prediction layer that concatenates the embeddings from different propagation layers and outputs the prediction score of a circRNA-miRNA pair.

#### 2.4.1 Embedding Layer

We use the embedding vector $e_{u_{i}}^{k}\in R^{s}\left(e_{v_{i}}^{k}\in R^{s}\right)$ to describe the circRNA *u* (miRNA *v*) in k-th layer, where *s* is the embedding size. The initial state of circRNA embeddings and miRNA embeddings in embedding layer can be abstracted as:

$$E_{u}^{0}=\left[e_{u_{1}}^{0},e_{u2}^{0},\ldots ,e_{u_{n}}^{0}\right]$$ (1)

$$E_{v}^{0}=\left[e_{v_{1}}^{0},e_{v2}^{0},...\;,e_{v_{m}}^{0}\right]$$ (2)

Where $E_{u}^{0}$ is the initial embedding of circRNAs, and $E_{v}^{0}$ is the initial embedding of miRNAs. The initial embedding will be continuously optimized and improved end-to-end, which will be mentioned in the next section.

#### 2.4.2 Embedding Propagation Layers

Next, we continuously aggregate the information of the node itself and its adjacent nodes to refine the embeddings of miRNAs and circRNAs. This is based on the GNN message-passing architecture (Hamilton et al., 2017; Xu et al., 2018). During an embedding update, the message aggregated by each node consists of two parts: the messages from the neighbor nodes of the previous layer and the messages inherited from the node itself.

**FIGURE 3:**
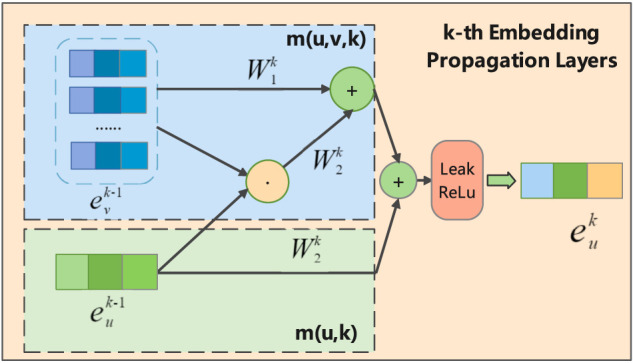
Illustration of message aggregation for circRNA u in k-th embedding propagation layer, where the $e_{v}^{k-1}$ represents the embedding of the neighbor node v of u in (k-1)-th layer.

As shown in Figure 3, in the k-th propagation layer, the embedding of circRNA *u* can be recursively formulated as:

$$e_{u}^{k}=\sigma \left(m\left(u,k\right)+\underset{v\in N_{u}}{\sum }m\left(u,v,k\right)\right)$$ (3)

Where $e_{u}^{k}$ represents the embedding of circRNA obtained in the k-th embedding propagation layer, *σ*(⋅) is the activation function LeakyReLU (Nikolakopoulos and Karypis, 2019), *v* denotes the neighbor nodes of u, and *m*(*u*, *k*) represent the messages delivered from the previous layer itself, while *m*(*u*, *v*, *k*) representing the messages delivered by all neighbor nodes from the previous layer. The *m*(*u*, *k*) and *m*(*u*, *v*, *k*) can be formulated as follows:

$$\begin{array}{c} m\left(u,k\right)=W_{1}^{k}e_{u}^{k-1} \end{array}$$ (4)

$$\begin{array}{c} m\left(u,v,k\right)=\frac{W_{1}^{k}e_{v}^{k-1}+W_{2}^{k}\left(e_{v}^{k-1}⊙e_{u}^{k-1}\right)}{\sqrt{N\left(u\right)\|N\left(v\right)|}} \end{array}$$ (5)

Where $W_{1}^{k},W_{2}^{k}\in R^{d_{k}\times d_{k-1}}$ are the trainable transformation matrices used to extract propagation information, and *d*_*k*_ is the transformation size; $e_{u}^{k-1}$ is the circRNA embedding representation generated from the (*k*−1)-th propagation layer, which will further contribute its information to the circRNA embedding *u* at layer *k*. We use the graph Laplacian norm $1/\sqrt{\left|N_{u}\right|\left|N_{v}\right|}$ to control how much the propagating message decays as the path length increases, where *N*(*u*) represent the first-hop neighbors of circRNA *u* (miRNA *v*). In Eq. 4, we consider the self-connection of nodes, which can effectively retain the original feature information to avoid information variation when the number of layers increases. For the neighbor nodes of node *u*, we aggregate not only the information of node *v* but also aggregate the interaction information between the *u* and *v*. It is encoded *via* $e_{v}^{k-1}⊙e_{u}^{k-1}$ , where ⊙ is element-wise product operation. In this way, more information from similar nodes can be passed, which enhances the representation ability of the model and helps to improve the accuracy of prediction results. Eqs 3–5 represent the calculation process of the embedding circRNA *u* at the *k*-th layer. Analogously, the embedded representation of miRNA can be obtained.

#### 2.4.3 Model Prediction

After multi-layer propagation, we can obtain multiple embedding representations of miRNAs and circRNAs. The embeddings obtained by different propagation layers contain different orders of interaction information, so they have different contributions to reflecting the relationship between circRNAs and miRNAs. Therefore, we concatenate all embeddings to express the final embedding. The following formula shows the final embedding representation of circRNA *u* and miRNA *v* through K embedding propagation layers:

$$e_{u}^{*}=e_{u}^{0}\|e_{u}^{1}\|\cdots \|e_{u}^{K},\;e_{v}^{*}=e_{v}^{0}\|e_{v}^{1}\|\cdots \|e_{v}^{K}$$ (6)

Where ‖ denotes concatenation operation, this simple concatenation operation can makes our final embeddings contain richer semantic information without increasing the learning parameters. Finally, we perform an inner product operation on the final embedding to obtain the interaction prediction between circRNA *u* and miRNA *v*:

$${\hat{y}}_{GCNCMI}\left(u,v\right)=e_{u}^{*}⊗e_{v}^{*}$$ (7)

Algorithm 1 shows the pseudocode description for predicting the interaction between circRNA *u* and miRNA *v* using GCNCMI.

#### 2.4.4 Model Optimization

Pointwise loss and pairwise loss are two common methods used to update model parameters (He et al., 2016). The pointwise learning emphasizes the loss between the predicted value ${\hat{y}}_{uv}$ and target value *y*_*uv*_. Still, we prefer to address predicting the interactions between circRNA and miRNA from the perspective of ranking. Therefore, we choose pairwise loss optimization to update model parameters. Bayesian Personalized Ranking (BPR) is a matrix factorization-based pairwise loss function that is often used to optimize recommendation tasks similar to our prediction task (Rendle, 2010). Specifically, it can be formulated as follows:

$$\underset{\Theta }{\min}L=\underset{\left(u,i,j\right)\in D}{\sum }-\ln s\left({\hat{y}}_{ui}-{\hat{y}}_{uj}\right)+\lambda \|\Theta {\|}_{2}^{2}$$ (8)

where *s*(⋯) is the sigmoid function; *D* = {(*u*, *i*, *j*)∣(*u*, *i*) ∈ R^+^, (*u*, *j*) ∈ R^−^} is the pairwise training sample containing positive samples R^+^(i.e., circRNA u has interacted with miRNA *v*_*i*_) and negative samples R^−^(i.e., the interactions between circRNA u and miRNA *v*_*j*_ is unknown). ${\hat{y}}_{ui}$ denotes the prediction score of u and *v*_*i*_. ${\hat{y}}_{uj}$ denotes the prediction score of u and *v*_*j*_. $\Theta =\left\{E,{\left\{W_{1}^{k},W_{2}^{k}\right\}}_{k=1}^{K}\right\}$ represents all model parameters that will be trained. *λ* is a parameter used to control the strength of *L*_2_ regularization. We use Adam as the optimizer to update the model parameters. Additionally, we use message dropout and node dropout to avoid model overfitting during training. Message dropout means that we will drop the message in Eq. 3 with a certain probability during the propagation, while node dropout randomly drops a specific node and discards all its outgoing messages. Dropout operations can reduce the influence of specific RNAs, making the model more robust.

Algorithm 1 GCNCMI algorithm to predict the interaction between circRNA u and miRNA v

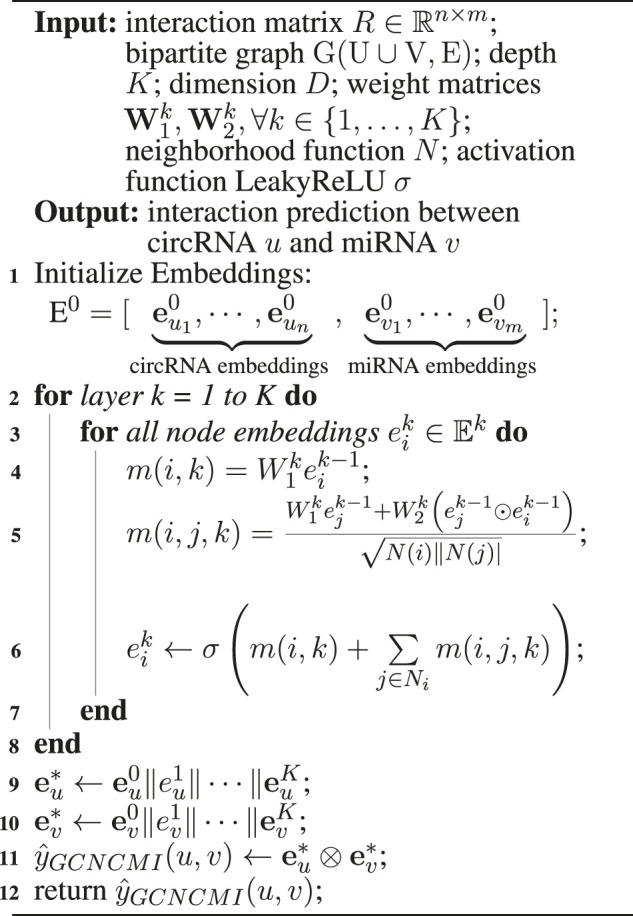

## 3 Experiment

### 3.1 Experimental Settings

To evaluate the performance of our model in predicting circRNA-miRNA interactions, we combined the known 9,589 interactions used as positive samples, and 9,589 unlabeled interactions were randomly selected from the benchmark dataset as negative samples. We performed five-fold cross-validation on the constructed dataset. The validated circRNA-miRNA interactions were randomly divided into five parts. Take each part as a positive sample and an equal number of unlabeled samples from the benchmark data as negative samples to form a test set. At the same time, perform the same operation on the remaining four parts to obtain a training set. This operation is performed until the loop is completed five times.

To measure the performance of GCNCMI more comprehensively, we used AUC, AUPR, Recall, Accuracy (Acc), precision (Pre), and F1 Scores. The definitions of each indicator are as follows:

$$Accuracy=\frac{TP+TN}{TP+TN+FP+FN}$$ (9)

$$Precision=\frac{TP}{TP+FP}$$ (10)

$$Recall=\frac{TP}{TP+FN}$$ (11)

$$F1=\frac{2*Precision*Recall}{Precision+Recall}$$ (12)

Where *TP* and *FP* represent the number of correctly classified samples and the number of misclassified samples in known circRNA-miRNA interactions, respectively, *TN* represents the number of correctly predicted unrelated circRNA-miRNA interactions, and *FN* represents the number of prediction errors in unrelated miRNA-circRNA interactions. *F*1 is a weighted average of model precision and Recall.

### 3.2 Cross-Validation Results

**FIGURE 4:**
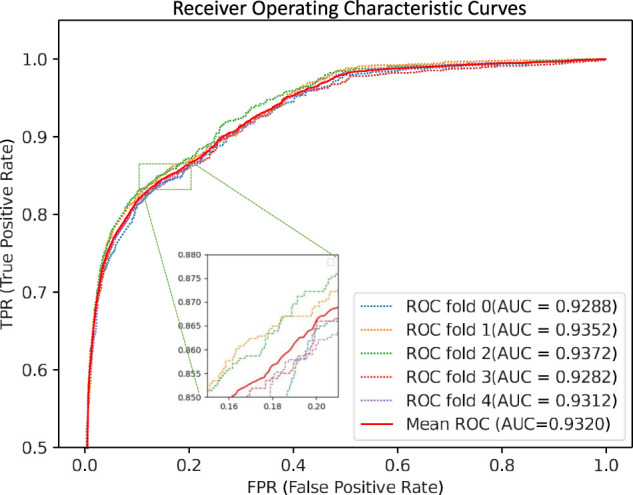
GCNCMI performed the ROC curves of five-fold cross-validation.

We performed five-fold cross-validations to evaluate the performance of the GCNCMI model in predicting circRNA-miRNA interactions. The experimental results of the five-fold cross-validation are shown in Table 2. As shown in the table, the AUC of the five-fold cross-validations are: 0.9288, 0.9352, 0.9372, 0.9282, 0.9312. On the AUPR, the AUPR of the five-fold cross-validations are 0.9293, 0.9428, 0.9453, 0.9396, 0.9412, respectively. In addition, we also plotted the ROC curve of GCNCMI, as shown in Figure 4. The above experimental results show that GCNCMI has good performance in predicting unknown circRNA-miRNA interactions.

**TABLE 2:** The five-fold cross-validation results of GCNCMI.

No.	AUPR	AUC	ACC	Pre	Recall	F1
1	0.9293	0.9288	0.8508	0.9390	0.8289	0.8805
2	0.9428	0.9352	0.8531	0.9424	0.8440	0.8905
3	0.9453	0.9372	0.8578	0.9450	0.8357	0.8870
4	0.9396	0.9282	0.8532	0.9392	0.8341	0.8835
5	0.9412	0.9312	0.8503	0.9408	0.8298	0.8818
Average	0.9396	0.9320	0.8530	0.9413	0.8345	0.8847

### 3.3 Parameter Influence

For GCNCMI, two essential parameters affect its performance: *K* (the number of layers) and *D* (the dimension of the embedding vector). When *K* is 2, and *D* is 256, our model GCNCMI achieves the best performance under five-fold cross-validation.

**FIGURE 5:**
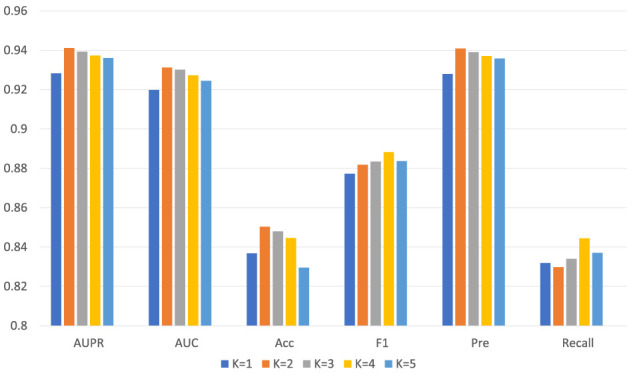
The performance of GCNCMI model on different layers.

The setting of the number of layers *K* indicates that our final embedding model incorporates the information of K-hop neighbor nodes in the bipartite graph, which can learn more hidden interaction information between nodes for the neural network. Table 3 lists the detailed values, and Figure 5 shows the trend chart for different layers. We tried from 1 to 5 layers for the number of layers of the model and found that the model’s accuracy at the beginning will increase with the increase of the number of layers. The best performance of the model is when the layer is 2. As the number of network layers increases, the hidden feature pairs of nodes tend to converge to the same value, which leads to an over-smoothing problem in the network.

**TABLE 3:** The performance of GCNCMI on different layers.

K	AUPR	AUC	Acc	Pre	Recall	F1
1	0.9283	0.9198	0.8368	0.9280	0.8319	0.8773
2	0.9412	0.9312	0.8503	0.9408	0.8298	0.8818
3	0.9393	0.9301	0.8480	0.9390	0.8340	0.8834
4	0.9374	0.9272	0.8446	0.9371	0.8444	0.8883
5	0.9361	0.9244	0.8295	0.9358	0.8371	0.8837

**FIGURE 6:**
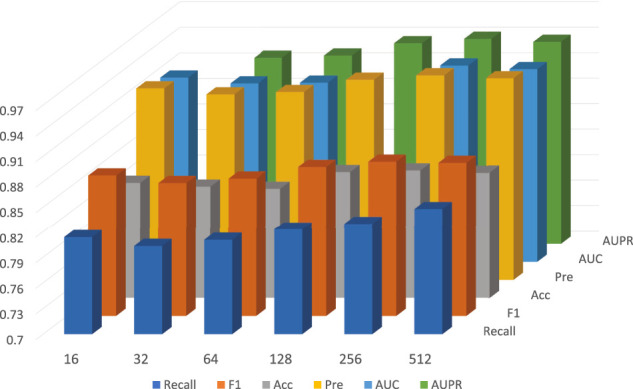
The performance of GCNCMI model on different embedding sizes.

On the other hand, under the framework of five-fold cross-validation, we conducted experiments for *D* in 16, 32, 64, 128, 256, 512, and other 6 cases; the detailed data is shown in Table 4. In general, as the dimension of the embedding vector increases, the expressive power of the model increases. But as can be seen from the Figure 6, from 16, 32, 64, 128, 256, the model’s performance has been increasing at first, but at 256, the commission has reached the maximum value. As *D* continues to grow, it will adversely affect the model’s performance.

**TABLE 4:** The performance of GCNCMI model on different embedding sizes.

D	AUPR	AUC	Acc	Pre	Recall	F1
16	0.9260	0.9170	0.8360	0.9257	0.8136	0.8660
32	0.9190	0.9102	0.8316	0.9187	0.8032	0.8571
64	0.9215	0.9110	0.8287	0.9212	0.8105	0.8623
128	0.9361	0.9265	0.8485	0.9357	0.8230	0.8757
256	0.9412	0.9312	0.8503	0.9409	0.8298	0.8819
512	0.9376	0.9268	0.8475	0.9373	0.8303	0.8806

### 3.4 Compared With State-Of-The-Art Methods

Since circRNA and miRNA interaction is a relatively new field, GCNCMI is the first method we know to predict the interaction between circRNA and miRNA, but other advanced methods in bioinformatics still provide us with reference. To better verify the performance of GCNCMI in inferring the interaction between circRNA and miRNA. We compare GCNCMI with six other state-of-the-art methods in bioinformatics.

Considering the scarcity of related biological resources, in calculating biological similarity, we only calculated Gaussian interaction profile biological similarity (GIP). In addition, since the adjacency matrix initialized each time is different, it requires us to re-mine the information in the bipartite graph. Strictly speaking, in similarity-based methods [AE-RF (Deepthi and Jereesh, 2021), KATZHMDA (Chen et al., 2017), NTSHMDA (Luo and Long, 2018)], the similarity matrix is recalculated each time during the cross-validation process. In the SDLDA method, we used SVD singular value decomposition to obtain linear features of circRNAs and miRNAs. The DMFMDA method chooses a Bayesian loss function over the loss function instead of the mean squared error.

**FIGURE 7:**
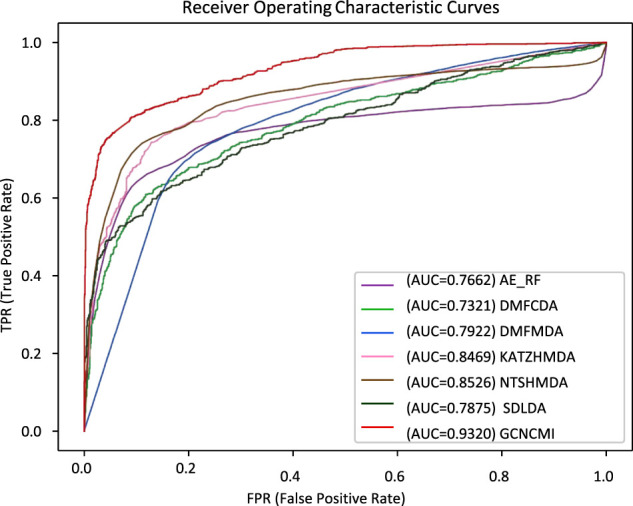
AUC values of different methods under five-fold cross-validation.

We performed a ten-times, five-fold cross-validation of GCNCMI with six advanced methods, changing the random number seed each time, and calculated the mean and standard deviation of 10 experiments. Table 5 lists several methods such as AE-RF (Deepthi and Jereesh, 2021), DMFCDA (Liu et al., 2020), DMFMDA (Liu et al., 2020), KATZHMDA (Chen et al., 2017), NTSHMDA (Luo and Long, 2018), SDLDA (Zeng et al., 2020), and compared with the GCNCMI model. Figure 7 plots the AUC curves to compare the seven methods. As can be seen from Table 5 and Figure 7, GCNCMI mines the high-order interactions between circRNA and miRNA; GCNCMI is higher than other methods in most indicators, among which the AUC value of GCNMCI is 0.9320, and the highest among different methods is NTSHMDA, whose AUC value is 0.8526, which is 7.94% lower than GCNCMI. GCNCMI value of AUPR is 0.9396, which is 6.24% higher than the second-best method, NTSHMDA. The above experimental results show that our model performs well in predicting the relationship between circRNA and miRNA.

**TABLE 5:** Performance comparison of different methods under five-fold cross validation.

Methods	AUC	AUPR	Acc	Pre	Recall	F1
AE-RF	0.7662 ± 0.0050	0.8239 ± 0.0042	0.8333 ± 0.0013	0.8923 ± 0.0019	**0.9279** ± 0.0019	**0.9097** ± 0.0010
DMFCDA	0.7321 ± 0.0240	0.7115 ± 0.0171	0.6975 ± 0.0112	0.8160 ± 0.0265	0.7729 ± 0.1112	0.7938 ± 0.0707
DMFMDA	0.7922 ± 0.0057	0.8230 ± 0.0089	0.7307 ± 0.0049	0.7030 ± 0.0080	0.7246 ± 0.0116	0.7136 ± 0.0065
KATZHMDA	0.8469 ± 0.0017	0.8647 ± 0.0019	0.8073 ± 0.0030	0.8511 ± 0.0055	0.7227 ± 0.0106	0.7816 ± 0.0071
NTSHMDA	0.8526 ± 0.0016	0.8772 ± 0.0018	0.6276 ± 0.0083	0.7556 ± 0.0518	0.4040 ± 0.0531	0.5264 ± 0.0486
SDLDA	0.7875 ± 0.0307	0.8286 ± 0.0189	0.6693 ± 0.0019	0.8287 ± 0.0108	0.7891 ± 0.0809	0.8084 ± 0.0706
GCNCMI	**0.9320** ± 0.0014	**0.9396** ± 0.0406	**0.8530** ± 0.0134	**0.9413** ± 0.0204	0.8345 ± 0.0301	0.8846 ± 0.0068

**FIGURE 8:**
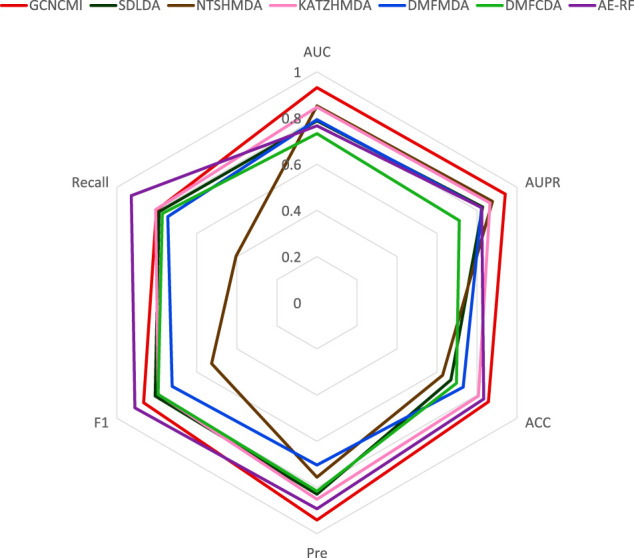
Radar plots of different methods on various performances.

The radar Figure 8 shows the performance of GCNCMI on AUC, AUPR, ACC, Recall, F1, Pre. The evaluation index is set from 0 to 1. As shown from Figure 8, the distance between the point and the center of the circle reflects the level of the value. It is evident that GCNCMI is better than other methods in predicting the circRNA-miRNA relationship.

**FIGURE 9:**
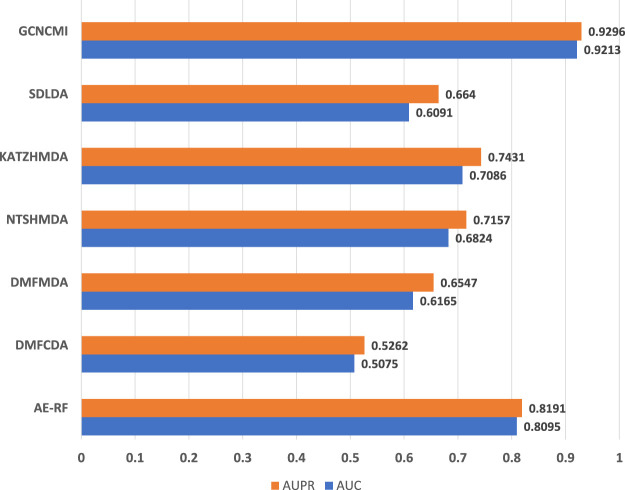
Comparison of AUC and AUPR values of GCNCMI and several other methods on independent test sets.

To further verify the accuracy of the GCNCMI model in circRNA-miRNA association prediction, we retrieved the data from the PubMed database, removed the known relationships that overlapped with the training dataset, and established a 9,386 miRNA-circRNA association relationship, 494 miRNAs, an independent test set of 1,502 circRNAs, and 9,386 unlabeled interactions were randomly selected from the benchmark dataset as negative samples. The specific information of the independent test set can be found in Table 6. Although there may be a small part of the independent test set and the unknown overlapping relationship in the training set, it can be ignored because it occupies a small proportion of the entire unvalidated sample set. The basic model for predicting circRNA-miRNA associations was obtained by training on our data set and tested on the independent test set. The test results are as Figure 9. The AUC of the GCNCMI model reached 0.9213, and the AUPR value reached 0.9296, which is higher than several other methods of comparison. The independent test results further showed that GCNCMI is an effective tool for inferring miRNA-circRNA associations.

**TABLE 6:** The number of circRNAs, miRNAs, and circRNA-miRNA interactions included in the independent test dataset.

circRNA	miRNA	interactions	unlabled interactions
1,502	494	9,386	9,386

### 3.5 Embedding Visualization

**FIGURE 10:**
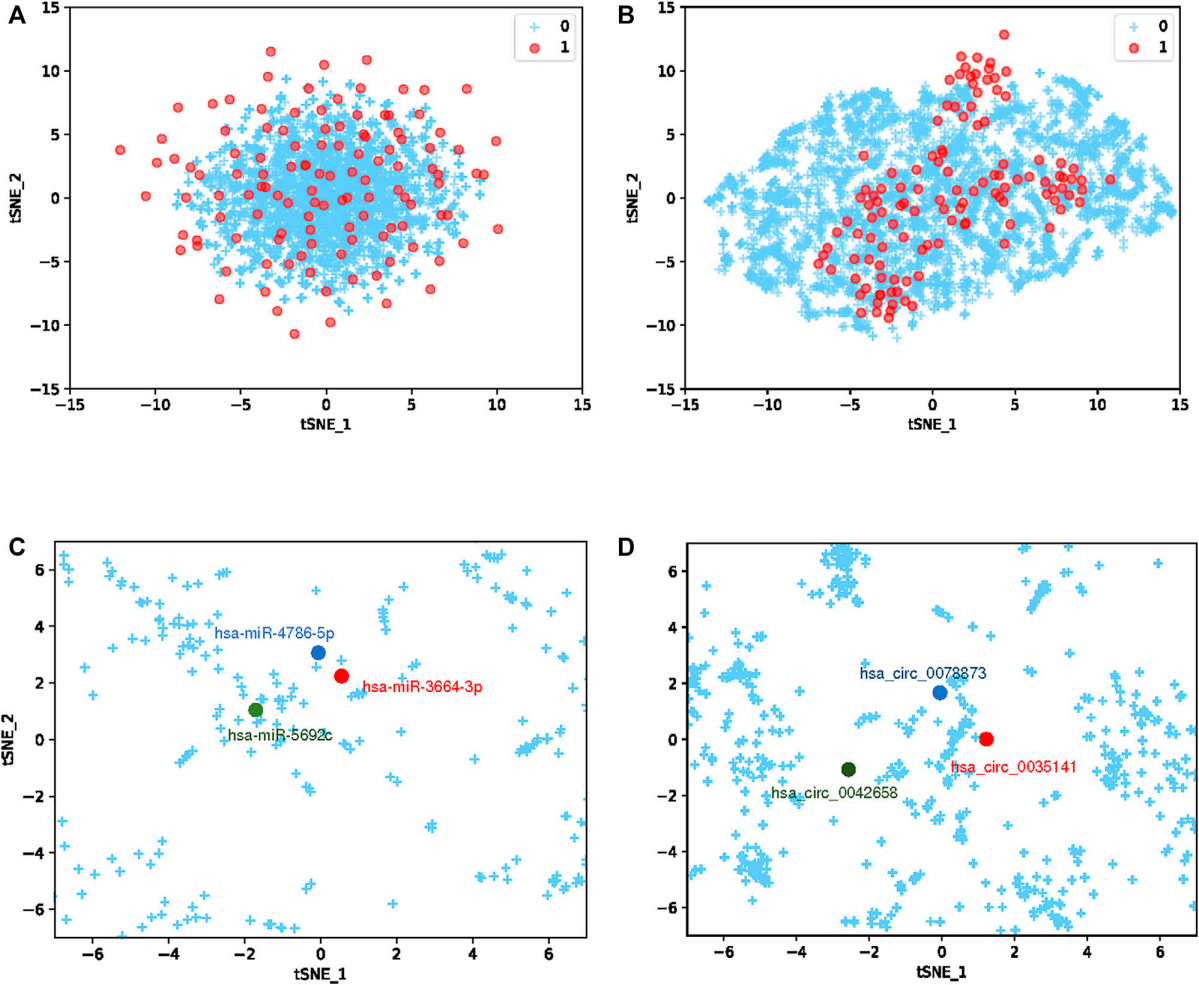
Embedding visualization **(A)** represents the embedding of the initialized circRNA-miRNA interaction pairs, and **(B)** represents the embedding representation of the circRNA-miRNA interaction pairs learned by the GCNCMI model. **(C)** represents the embedding of miRNA after learning by the GCNCMI model, and **(D)** represents the embedding of circRNA after learning by the GCNCMI model.

To more clearly demonstrate the learning ability of the GCNCMI, We use T-SNE (Van der Maaten and Hinton, 2008) to visualize the embedding of circRNA-miRNA interaction pairs. Because the number of unknown relationships is much larger than the number of known associations, and to better visualize the overall mining of higher-order relationships by GCNCMI, we choose to visualize more unlabeled samples than labeled samples. The main goal of T-SNE is to convert multi-dimensional datasets into low-dimensional datasets. Compared with other dimensionality reduction algorithms, T-SNE is the most effective technique in data visualization. Since T-SNE is not a linear dimensionality reduction technique, it can capture the complex manifold structure of high-dimensional data. We initially a 32-dimensional vector to represent miRNA and circRNA. To explore the similarity between vector representations, we used the T-SNE algorithm to reduce the vector to 2-dimensional, as shown in Figure 10A. The blue + represents unknown miRNA-circRNA interaction pairs, and the red dots represent the known circRNA-miRNA interaction pairs. Figure 10B shows the embedding of the circRNA-miRNA interactions learned by the GCNCMI model. Comparing Figures 10A,B, it can be seen that GCNCMI has a good effect on mining high-order interactions between miRNAs and circRNAs, and the GCNCMI can better use the known interaction pairs to mine potential miRNA-circRNA interaction pairs. In addition, we also visualized the learned circRNA embeddings and miRNA embeddings. Figure 10C shows the learned miRNA embeddings. We used the GCNCMI model to predict the top 30 circRNAs most closely associated with each miRNA, and also predicted the top 30 miRNAs most closely associated with each circRNA. The hsa-miR-4786-5p and hsa-miR-3664-3p were associated with nine similar circRNAs, and hsa-miR-4786-5p and hsa-miR-5692c were associated with five similar circRNAs. Therefore, the hsa-miR-4786-5p is more similar to hsa-miR-3664-3p. It can also be seen from Figure 10C that the distance between hsa-miR-4786-5p and hsa-miR-3664-3p is closer. Figure 10D shows the visualization of the embedding of circRNAs after model learning. The hsa-circ-0078873 and hsa-circ-0042658 were associated with three similar miRNAs, and hsa-circ-0035141 and hsa-circ-0078873 were associated with seven similar miRNAs. Therefore, hsa-circ-0078873 is closer to hsa-circ-0035141, and it can be seen from Figure 10D that hsa-circ-0078873 is closer to hsa-circ-0035141. The experimental results show that GCNCMI can effectively learn the potential higher-order interactions between miRNAs and circRNAs.

### 3.6 Case Studies

It is of great significance to discover unknown associations between circRNAs and miRNAs. We selected two miRNAs, hsa-miR-622 and hsa-miR-149-5p, for case studies. Specifically, we first delete the circRNAs that have been experimentally validated for the selected miRNAs. Then, the remaining circRNAs were sorted in descending order according to the values predicted by the GCNCMI model. The following shows the results of the normalized prediction scores of the GCNCMI model. Finally, we screened the top 10 circRNAs and collected evidence in the published literature for testing.

miR-622 (Lu et al., 2022) is a miRNA of 13q31.3 in the eukaryotic genome, and its expression is mainly in the nucleus. In recent years, studies have found that miR-622 can functionally inhibit the malignant proliferation of cells, which is helpful for cancer treatment. In recent years, miR-149-3p (Yang et al., 2017) can effectively inhibit the proliferation and apoptosis of malignant tumors. Recent studies have found that miR-149-3p can increase the sensitivity of drugs. Table 7 and Table 8 list the top 10 candidate circNRAs of hsa-miR-622 and hsa-miR-149-5p. We selected the top 10 candidate circRNAs as our predicted circRNAs, respectively, and finally, we compared the predicted results with the experimentally validated interactions. It can be seen that 7 of hsa-miR-622 were confirmed by existing evidence, and 8 of hsa-miR-149-5p were confirmed by existing evidence. It should be noted that unproven associations may exist and require further experimental verification.

**TABLE 7:** The top 10 circRNAs with the closest relationship to hsa-miR-622 predicted by GCNCMI model.

Rank	CircRNA	Evidence(PMID)	Score
1	hsa_circ_0000231	34183076	0.8822
2	hsa_circ_0101432	Unconfirmed	0.8820
3	hsa_circ_0119872	33579337	0.8815
4	hsa_circ_0008574	32616043	0.8798
5	hsa_circ_0000211	31668923	0.8796
6	hsa_circ_0001273	35567340	0.8712
7	hsa_circ_0086902	Unconfirmed	0.8592
8	hsa_circ_KCNQ5	35413218	0.8542
9	hsa_circ_0101432	35297300	0.8498
10	hsa_circ_0006000	Unconfirmed	0.8469

**TABLE 8:** The top 10 circRNAs with the closest relationship to hsa-miR-149-5p predicted by GCNCMI model.

Rank	CircRNA	Evidence(PMID)	Score
1	hsa_circ_0061140	32224273	0.8737
2	hsa_circ_0075341	31706100	0.8722
3	hsa_circ_0008956	34153672	0.8702
4	hsa_circ_0000654	31778020	0.8693
5	hsa_circ_0051239	Unconfirmed	0.8689
6	hsa_circ_ROBO2	34649241	0.8673
7	hsa_circ_0011385	34720052	0.8672
8	hsa_circ_0087352	35286916	0.8671
9	hsa_circ_0123996	32707301	0.8661
10	hsa_circ_0031059	Unconfirmed	0.8648

## 4 Conclusion

CircRNAs are circular non-coding RNAs with regulatory functions, most of which exist in eukaryotic excerpts, and most circRNAs are composed of exons. Because circRNAs are less affected by nucleases, circRANs are more stable than linear RNAs. Current studies have shown that circRNAs can competitively adsorb miRNAs, and circRNAs can bind to proteins to inhibit the activity. Therefore, there is an urgent need to explore the relationship between circRNA and miRNA. However, because traditional biological experiments are time-consuming and labor-intensive, a more efficient method is needed to explore the potential relationship between circRNA and miRNA.

In this paper, we proposed a graph convolutional neural network prediction model for circRNA and miRNA interactions. To fully exploit the potential high-order interactions between circRNAs and miRNAs, we designed a graph convolutional neural network method to propagate the interaction’s relation recursively without computing the similarity of circRNAs and miRNAs. The experimental results demonstrated the excellent performance of GCNCMI in predicting the interactions between circRNAs and miRNAs. The results of independent tests indicate that the GCNCMI model has good generalization performance in predicting unknown circRNA and miRNA relationships. Finally, a case study compared our predictions with those validated by biological experiments, further demonstrating the model’s excellent predictive performance. The above results indicate that GCNCMI is an excellent method for predicting the potential interactions between circRNAs and miRNAs.

While GCNCMI has excellent performance, it also has some limitations. First, due to the scarcity of biological resources, GCNCMI only uses the association data of circRNAs and miRNAs, and the quality of the data will affect the performance of GCNCMI model training. In the future, using heterogeneous data from multiple perspectives will be considered to improve the model’s performance further.

## Data Availability

The original contributions presented in the study are included in the article/Supplementary Material, further inquiries can be directed to the corresponding author.
