# Supplementary material for: GCNCMI: A Graph Convolutional Neural Network Approach for Predicting circRNA-miRNA Interactions
Source: Front Genet. 2022 Aug 5;13:959701. doi: 10.3389/fgene.2022.959701 (PMC9389118; doi:10.3389/fgene.2022.959701)
Supplement: Supplementary file 1 [file DataSheet1.zip › YM-logo.pdf]

frontiers FOR YOUNG MINDS
