# Supplementary figures and images for: GCNCMI: A Graph Convolutional Neural Network Approach for Predicting circRNA-miRNA Interactions
Source: Front Genet. 2022 Aug 5;13:959701. doi: 10.3389/fgene.2022.959701 (PMC9389118; doi:10.3389/fgene.2022.959701)

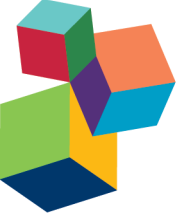

frontiers

Supplement: Supplementary file 1 [file DataSheet1.zip › logo1.pdf]

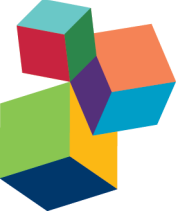

Supplement: Supplementary file 1 [file DataSheet1.zip › logo2.pdf]

A

frontiers  
FOR YOUNG MINDS

B

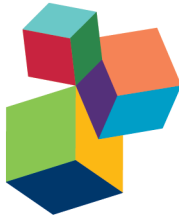

Supplement: Supplementary file 1 [file DataSheet1.zip › logos.pdf]

# Receiver Operating Characteristic Curves

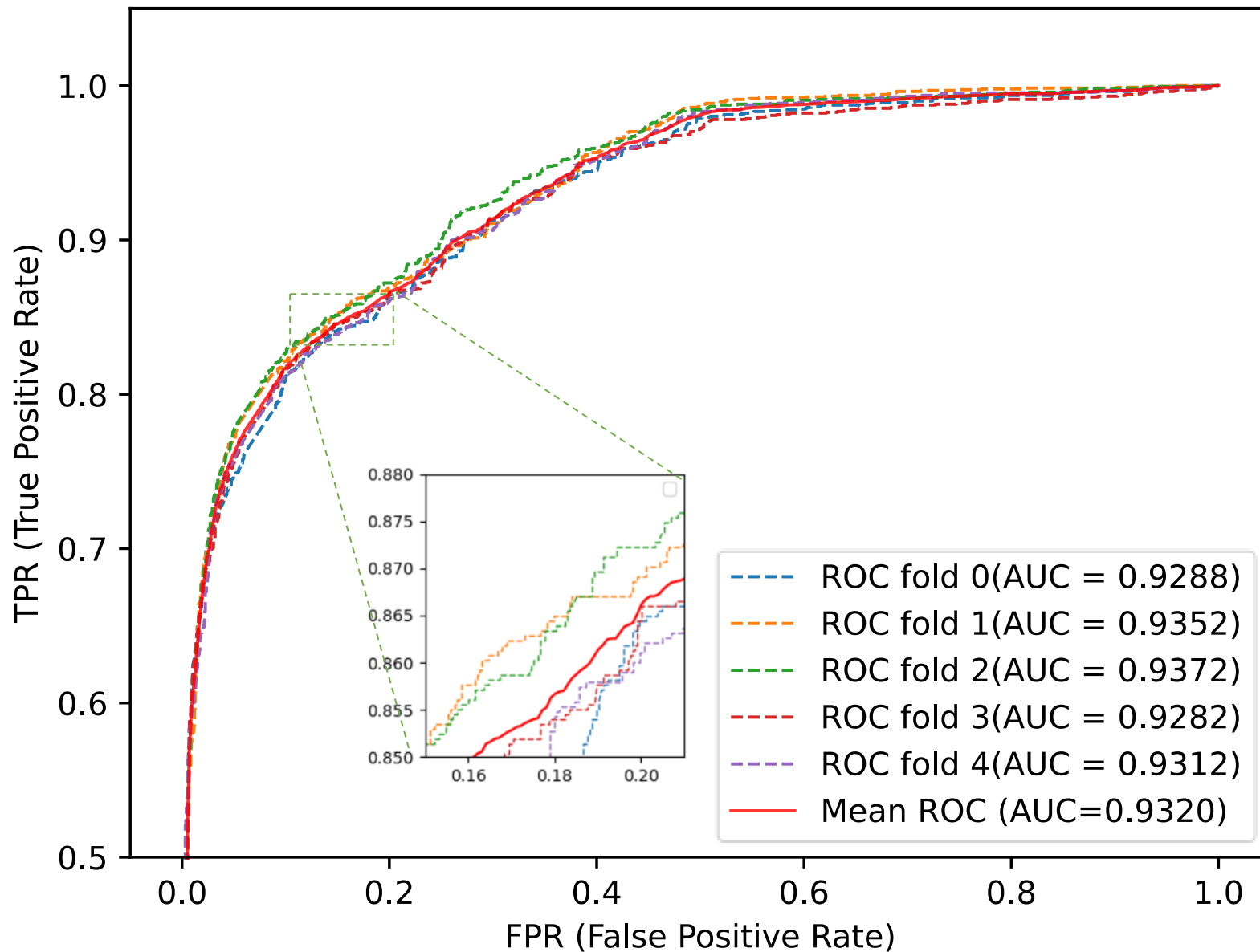

Supplement: Supplementary file 1 [file DataSheet1.zip › fold-auc-333Curve.pdf]
